# Par2-mediated responses in inflammation and regeneration: choosing between repair and damage

**DOI:** 10.1186/s41232-024-00338-1

**Published:** 2024-05-30

**Authors:** Gal Reches, Ron Piran

**Affiliations:** The Azrieli Faculty of Medicine, Bar-Ilan University, 8 Henrietta Szold St, Safed, Israel

**Keywords:** Protease-activated receptor-2 (Par2), Treatment, Inflammation, Injury, Regeneration, Healing

## Abstract

The protease activated receptor 2 (Par2) plays a pivotal role in various damage models, influencing injury, proliferation, inflammation, and regeneration. Despite extensive studies, its binary roles— EITHER aggravating injury or promoting recovery—make a conclusive translational decision on its modulation strategy elusive. Analyzing two liver regeneration models, autoimmune hepatitis and direct hepatic damage, we discovered Par2’s outcome depends on the injury’s nature. In immune-mediated injury, Par2 exacerbates damage, while in direct tissue injury, it promotes regeneration. Subsequently, we evaluated the clinical significance of this finding by investigating Par2’s expression in the context of autoimmune diabetes. We found that the absence of Par2 in all lymphocytes provided full protection against the autoimmune destruction of insulin-producing β-cells in mice, whereas the introduction of a β-cell-specific Par2 null mutation accelerated the onset of autoimmune diabetes. This pattern led us to hypothesize whether these observations are universal. A comprehensive review of recent Par2 publications across tissues and systems confirms the claim drafted above: Par2’s initial activation in the immune system aggravates inflammation, hindering recovery, whereas its primary activation in the damaged tissue fosters regeneration. As a membrane-anchored receptor, Par2 emerges as an attractive drug target. Our findings highlight a crucial translational modulation strategy in regenerative medicine based on injury type.

## Introduction

The intricate role of the protease-activated receptor 2 (Par2) activation, catalyzed primarily by trypsin, has captivated researchers due to its profound impact on biomedical responses and its implications in various diseases and conditions, particularly inflammation and regeneration processes. As a membrane-bound receptor involved in crucial cellular signaling pathways, Par2 has emerged as an enticing target for therapeutic intervention, igniting substantial interest within the scientific community. Par2 is known to regulate pain signaling and swelling [1–4], inflammatory bowel disease [5–7], pulmonary and asthmatic reactions [8–11], pancreatitis [12–14], as well as skin irritation and itch [15–18]. Furthermore, potent Par2 agonists and antagonists have been developed over the years and successfully tested in animal models [4, 5, 10, 11, 19–22]. However, the perplexing nature of Par2 activation has impeded its translation into clinical applications, as the observed experimental outcomes of Par2 activation are puzzling, EITHER aggravating or alleviating the disease phenotype, sometimes within the same tissue. In this study, we present a comprehensive analysis of Par2-mediated cell responses and propose a novel treatment strategy for different disease types.

Our investigations into Par2 activation have revealed intriguing observations in models of liver damage. When exposed to concanavalin A (ConA) poisoning, Par2 led to significant hepatitis with marked leukocyte infiltrations, while Par2 knockout (Par2KO) mice showed protection against liver damage without infiltrates [23]. Surprisingly, when carbon tetrachloride (CCl_4_) replaced ConA, we noticed the exact opposite phenotype: wild-type (WT) mice exhibited rapid recovery from liver damage, whereas Par2KO mice failed to heal. To elucidate these opposing effects, we conducted reciprocal bone marrow (BM) replacement experiments to isolate the immune system from the affected organ. In the ConA model, BM replacement showed that hepatocellular damage correlated with Par2 expression in the immune system, while in the CCl_4_ model, hepatic recovery depended on Par2 expression in the damaged tissue rather than the immune system.

The ConA model damage is mediated by T-lymphocyte-induced hepatocellular damage [24, 25], while in the CCl_4_ model, hepatocellular injury is directly generated as a result of free radical formation [12, 26, 27].

Our findings led us to conclude that the role of Par2 in aggravating or alleviating the damage is determined by the tissue in which it is first activated. We concluded that when Par2 was activated in the immune system (as in the ConA model), it aggravated inflammation, whereas when it was activated in the damaged tissue using CCl_4_, it promoted regeneration.

We assessed the clinical implications of this observation by analyzing Par2’s expression in the context of autoimmune diabetes. We distinguished between the two opposing arms of autoimmune conditions present in real disease: the immune arm, which destroys tissue, and the regenerative arm, which acts to restore it. We engineered tissue-specific Par2 null mutations as Tm1c [28], in the immune system, β-cells, and in the eye retina (as a control). We introduced these mutations into the NOD mouse, the prominent model of type I diabetes. The results of these manipulations were remarkable. Complete Par2 deficiency in all lymphocytes provided mice with full protection against autoimmune destruction of β-cells, whereas the β-cell specific Par2 mutation accelerated the onset of autoimmune diabetes. These findings underscore the potential benefit of Par2 modulation in autoimmune conditions in general, particularly type I diabetes [29].

Building upon these results, we sought to investigate whether this phenomenon extends beyond hepatic tissue and holds true across different injury and recovery models. We hypothesized that the conclusion drafted above is universal to Par2-mediated phenotypes.

Therefore, after a brief revisit of Par2’s mode of action and biochemistry, we conducted a comprehensive study of the recent literature on Par2 signaling to test our hypothesis. By thoroughly analyzing the recent literature on Par2 signaling, we divided the findings based on the distinction between inflammatory processes propagating the damage and direct tissue injury. Our extensive research corroborated our hypothesis. We did not find any work that contradicts it and thus provided evidence that Par2 modulation could serve as a complementary approach to address various injuries, induce tissue regeneration, and promote recovery.

### The challenges of targeting Par2

Pars are a group of four members within the G-protein-coupled receptor (GPCR) superfamily, comprising a seven-transmembrane structure, while the intracellular domain typically binds to different Gα and βγ subunits that transmit the internal signal. Common transmembrane receptors usually have ligands, and their activity is regulated by the formation of the ligand-receptor complex, which induces a conformational change in the cytosol-facing part of the receptor, thereby activating the signal. Protease-activated receptors, as implied by their names, are activated by proteolytic cleavage of a residue from the receptor itself (N-terminal), rendering the receptor active. Once activated, the receptor remains constitutively active, and there is no direct biochemical way to terminate the signal, apart from internalizing and degrading the receptor.

### Par2 mechanism of action

**Fig. 1:**
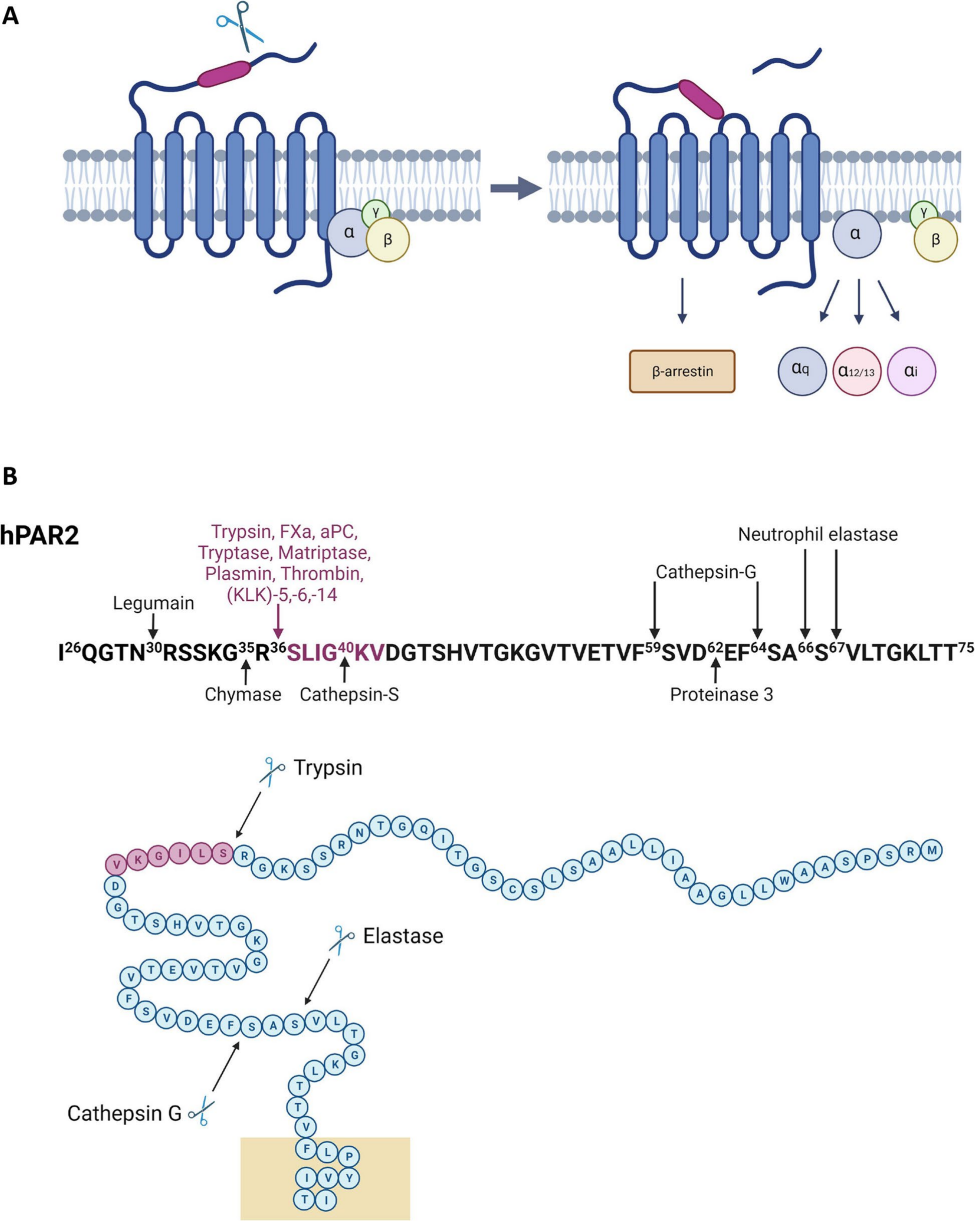
Par2 structure and biochemical activation.** A** Activation of Par2 occurs through cleavage of its N-terminal region, which exposes the tethered ligand domain. The exposed ligand then binds to the receptor’s other extracellular loops, initiating intracellular signalling pathways. These pathways include recruitment of G-proteins and β-arrestin. **B** Human Par2 N-terminus: major activating proteases and cleavage sites are indicated. The amino acid residues highlighted in purple represent the canonical tethered ligand

Par2 activation involves proteolytic cleavage at the N-terminal region, exposing a peptide sequence known as the tethered ligand (TL). This TL binds intramolecularly to Par2, activating the receptor and initiating intracellular signal transduction [30]. Par2 exhibits diverse signaling pathways mediated by different G protein α subunits, including Gαq/11, Gαi, Gα12/13, and Gαi/o [3, 31] (Fig. 1A). Besides G protein-dependent pathways, Par2 can initiate G protein-independent signaling via β-arrestin recruitment to its carboxy-terminus [3, 32, 33]. Following trypsin activation, Par2 undergoes receptor phosphorylation, regulating its cell surface localization. Constitutive (by protease) and agonist-induced activation lead to receptor internalization and eventual degradation to terminate its signaling [3, 32, 34–38]. Par2 activation leads to various downstream effects, including calcium influx through Gαq, increased cAMP levels via Gαs, enhanced Rho-Kinase activity mediated by Gα12/13 [39, 40], increased Rho-Kinase activity by Gα12/13 [41], recruitment of β-arrestin-1 and β-arrestin-2 [33], and ERK1/2 phosphorylation [37, 42], ultimately culminating in Par2 internalization and degradation [33, 36, 40, 43–49].

Recent studies have identified a repertoire of proteases that cleave Par2, either at the trypsin recognition site (canonical activation) by proteases such as tryptase [50–52], matriptase [53, 54], FXa, FVIIa [54–56], plasmin [57, 58], thrombin [57, 59], and kallikrein (KLK)-5,-6,-14 [60], or at different cleavage sites within the PAR2 sequence (known as noncanonical activation), leading to the generation of distinct tethered ligands by enzymes like proteinase-3, cathepsin-G [61], granzymes [62], legumain [63], and KLKs [64, 65]. These cleavage events can result in different cellular processes than those triggered by canonical pathway activation [39]. Furthermore, Par2 can undergo receptor inhibition through complete removal of the activating tethered ligand, a process referred to as disarming or shedding [3, 31, 39] (Fig. 1B). Additionally, emerging evidence suggests interplay between Pars, with transactivation or coactivation processes occurring, such as Par1 tethered ligand activating Par2 in cells expressing both receptors [31].

While the biochemical pathways and signaling mechanisms of Par2 have been extensively studied, understanding the different cellular as well as organismal phenotypes resulting from the activation of these same pathways in different tissues and cell types has remained a challenge. These contradictory observations have impeded the inclusion of Par2 modulation as a viable treatment strategy for many years.

**Fig. 2:**
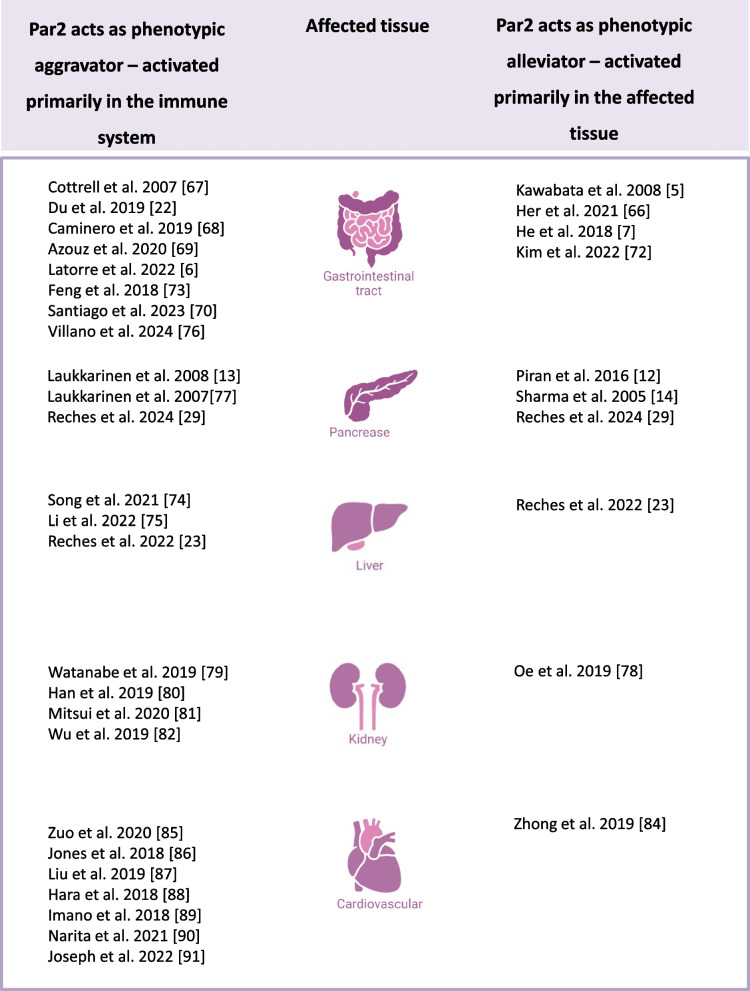

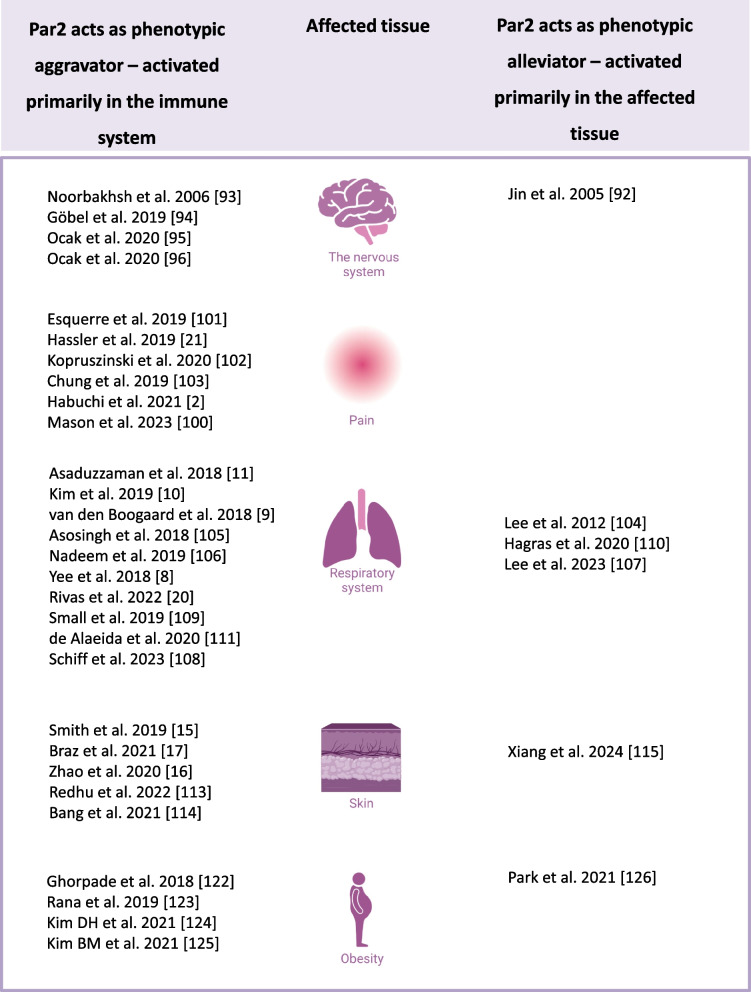
Par2 perplexing effects in the same tissues. Par2 studies in different tissues, categorized based on the observed phenotype. In studies where the damage is immune-mediated, Par2 exhibits an aggravating role (left column). Conversely, in studies where the damage is a result of direct injury, Par2 activation alleviates the damage (right column)

In the subsequent sections, we provide a comprehensive revisit of the contradicting phenotypes associated with Par2 activation in different tissues, damage models, and view these findings through the lens of our hypothesis. These studies are found to confirm our hypothesis that Par2-induced damage occurs when activated by the immune system, and Par2-induced regeneration occurs when activated by the injured tissue (Fig. 2).

### The gastrointestinal tract

In the gastrointestinal (GI) tract, Par2 has been found to exert protective effects against gastric injuries induced by acid or ethanol [5]. In addition, deletion of Par2 has been shown to exacerbate colitis and impair colonic regeneration in response to a high-fat diet [66]. However, Par2 deletion protected animals from enteritis induced by *Clostridium difficile* [67], and treatment with a Par2 antagonist provided protection from inflammatory bowel disease (IBD) induced by *Trichinella spiralis* [22]. Moreover, Par2 activation aggravated inflammation induced by *Pseudomonas aeruginosa* [68]. Adding to the confusion, in the esophagus, another section of the GI, Par2 inhibition was found to reduce Eosinophilic esophagitis both in vivo and in vitro [69].

When inflammatory bowel disease (IBD) was induced by dextran sodium sulfate (DSS), Par2KO hampered efficient recovery, indicating that Par2 is an important mediator of gut regeneration [7]. However, in another study combining DSS and trinitrobenzene sulphonic acid (TNBS), inflammation and pain were induced by Par2 signaling [6]. Recently, in a mouse model of Crohn’s disease, a novel Par2 cleavage-resistant mouse was utilized [70]. It was discovered that the proteolytic activity induced by microbiota isolated from Crohn’s patients exhibits proinflammatory properties, exacerbating colitis severity in germ-free mice by activating the PAR2 pathway.

These seemingly contradictory roles of Par2 can be explained when considering the PAR2 bi-modular theory presented above. Positive effects of Par2 on healing and regeneration are observed in models of direct injury where inflammatory processes have a minor role [5, 7, 66]. In contrast, immune-mediated damage models, such as exposure to pathogens and allergens, result in Par2 activation playing a deleterious role [22, 67–69]. This observation is supported by two seemingly contradicting works that were conducted in cell lines: Par2 activation in the Caco2 cell line, derived from a human colorectal carcinoma and widely used as an intestinal cell line [71], enhanced tight junctions and decreased mucosal permeability, indicating a protective role of Par2 in the gut epithelium [72]. However, in a study conducted in lymphocytic T-helper cells from patients with ulcerative colitis, Par2 was found to mediate an inflammatory response [73].

### The liver

In a mouse ischemia–reperfusion model, Song et al. demonstrated that Par2 participates in and aggravates mast cell-induced inflammatory response. Mast cells secrete tryptase, a Par2 activator, and indeed, injury was markedly reduced in mast cell-deficient mice [74]. In 2022, Li et al. reported that the hepatitis B virus X protein (HBVX) induces hepatitis, and this inflammatory response is mediated by Par2. In Par2KO mice, the HBVX response was dramatically alleviated [75]. In a diet-induced non-alcoholic steatohepatitis (NASH) model, treatment with 1-PPA, a recently identified Par2 inhibitor, effectively suppressed the PAR2—C/EBP-β—SerpinB3 axis, resulting in protection against NASH development and progression [76].

We recently compared two hepatitis models, one induced by CCl_4_ and the other by ConA. It was found that Par2 plays different roles depending on whether it is expressed in immune cells or hepatic tissue. CCl_4_ induces hepatitis via direct injury to the hepatic tissue, while ConA, a lectin that is presented on MHC molecules, induces hepatic injury that is mediated by the immune response. In a series of BM replacement experiments, we separated immune system Par2 from Hepatic Par2, generating two complementary strains. One expressing Par2 in the liver, with no Par2 expressed in leukocytes, and the other being the exact mirror image. In the ConA model, we demonstrated that when Par2 was expressed in the immune system, the inflammatory response was intensified, as shown by Song et al. and Li et al. However, when Par2 was expressed in the tissue and not in the immune cells, the livers were protected from the damage induced by ConA. However, when we attempted to replicate these findings in the CCl_4_ model, we found that immune cell Par2 did not affect the damage, and liver recovery was completely dependent on Par2 expression in hepatocytes [23]. These observations led us to the conclusion that Par2 has two roles: in leukocytes, it promotes an inflammatory response, and in the target tissue, it promotes regeneration.

### The pancreas

We previously demonstrated that healing from acute pancreatitis induced by Caerulein required Par2, as Par2KO mice failed to recover. Moreover, the pancreatic damage was not limited to the exocrine pancreas as in Par2KO mice the endocrine tissue also degenerated following Caerulein [12]. However, in another model of pancreatitis, in which a retrograde pancreatic ductal infusion of bile salts was used, pancreatitis was intensified in WT animals while Par2KO mice were largely protected [13, 77]. Again, these confusing observations can be reconciled as Caerulein acts directly on the pancreas, initiating massive secretion of digestive enzymes. However, bile salts are known to activate the immune system, primarily eosinophil degranulation [74].

To prove the hypothesis of Par2 bi-modular theory, we employed tissue-specific Par2 null mutations targeting the immune system, β-cells, and the retina in the type I diabetic NOD mouse model. The outcomes of these genetic modifications were clear. Total Par2 deficiency in lymphocytes conferred complete protection against autoimmune β-cell destruction, while the β-cell-specific Par2 mutation hastened the onset of autoimmune diabetes [29].

### The kidney

Vascular endothelial growth factor (VEGF) plays a propagative role in several cancer types. Therefore, antagonizing VEGF is an important approach in treating these cancers. However, many patients who receive VEGF-antagonizing treatment suffer from kidney disease. In an attempt to model kidney disease induced by VEGF antagonizing antibody, it was found that in mice, this phenotype is induced only when the endothelial nitric oxide synthase (eNOS) gene is mutated [78]. When an antagonizing antibody to VEGF was administered, Par2 and eNOS double mutants developed severe kidney disease characterized by glomerular damage compared to eNOS single mutants. This indicates that Par2 acts to alleviate kidney disease.

Cisplatin, a chemotherapy drug, may also induce kidney injury. In this case, Par2KO mice were protected from kidney injury induced by cisplatin [79]. Consistent with these findings, Par2 was found to aggravate kidney injury induced by nephrotoxic serum nephritis (NTN) as Par2 antagonist treatment reduced glomerular crescent formation and thrombosis [80]. It is worth mentioning a study that involved the inhibition of both Par1 and Par2 to target diabetic kidney disease, indicating that Par2 acts to aggravate this complication of diabetes [81].

It appears that the first work on VEGF antagonists is an outlier regarding Par2’s role in kidney disease. However, when one revisits our hypothesis, which states that if Par2 is activated in the tissue, it promotes regeneration, while if it is activated in the immune system, it aggravates inflammation, these reports do not contradict at all. The VEGF-antibody complex induces direct injury in the nephron, and thus regeneration is stimulated by Par2 [78]. The other reports indicate a leukocyte infiltration phenotype, indicating an immune reaction in the damage model. Therefore, Par2 inhibition either prevented infiltration formation or inhibited its accumulation [79–81].

In relation to the reports above, urinary tract inflammation induced by *E. coli* is aggravated via Par2 activation by tryptase mast cells [82].

A study conducted by Ma et al. seems to contradict our hypothesis. In their work, they induced direct kidney injury by obstructing the lateral ureteric duct [83]. Par2 deletion was not accompanied by any alleviation or aggravation of the phenotype. According to the hypothesis in which Par2 in the tissue promotes regeneration, one would expect to see a regenerative advantage in the WT. However, it should be noted that the irreversible nature of the obstruction and the lack of post-damage opening of the ligation precluded the observation of regeneration. We hypothesize that if the ligation had been removed, a regenerative advantage would have been observed in WT animals compared to Par2KO animals.

### The cardiovascular system

Par2 has shown a protective role in myocardial ischemia–reperfusion injury in a short duration damage model, where the left anterior descending artery (LAD) was ligated for 45 min and then released for reperfusion. In this model, Par2 exhibited a protective role on the heart [84]. Conversely, in a model of permanent myocardial infarction, where the LAD was ligated for 28 days, hematopoietic Par2KO reduced myocardial inflammation, macrophage infiltration into the myocardium, and pro-inflammatory cytokine levels. This effect was achieved through Par2KO BM reconstitution in irradiated WT mice [85]. The apparent contradiction between these two studies can be reconciled by considering the nature of the damage. In the short ligation model, where the suture was removed, the damage is a direct injury, and Par2 activation plays an alleviatory role. However, in the permanent ligation model, the lack of tissue oxygen and nutrients due to the blocked artery leads to tissue necrosis and inflammation, preventing regeneration. Therefore, Par2’s prominent role in this context is in the immune system.

In a genetic mouse model of atherosclerosis with LDL receptor mutation, Par2 deficiency was found to attenuate the disease [86]. Similarly, in another atherosclerosis model combining ApoE gene mutation and tissue factor overexpression, Par2 antagonist reduced inflammation, intra-plaque hemorrhage, and neovascularization. This phenotype can be explained from both the leukocytes’ Par2 inhibition—reduced inflammatory markers, and from the epithelial-cells’ Par2 inhibition—inhibited neovascularization, which is a regenerative response by these cells [87]. Furthermore, a study combining ApoE deletion with BM replacement experiments demonstrated that Par2KO mice exhibited a decreased atherosclerotic phenotype compared to control mice [88]. This study indicated that the atherosclerotic phenotype is mediated by Par2 activation in the immune system, which aligns with our recent work demonstrating the role of leukocyte Par2 in ConA-induced hepatitis [23].

In a different damage model involving clot formation, intermittent hypoxia for 28 days increased inflammatory markers and tissue degeneration. Par2 antagonist administration reduced these phenotypes [89]. Similarly, in a study inducing hypertension by renin overexpression, which led to inflammation and tissue scar formation, Par2 antagonist alleviated inflammation and fibrosis. As in the former study, Par2 antagonist on the renin overexpression background was able to ameliorate these inflammation and fibrosis [90].

Another study on pulmonary arterial damage found that Par2KO mice were protected from chronic hypoxia-induced damage, exhibiting reduced vascular muscularization of small pulmonary arteries and other inflammatory markers [91].

### The nervous system

In a direct injury model of transient focal cerebral ischemia, where intraluminal middle cerebral artery blockade was induced for 1 h, Par2 was found to induce rapid healing, as the severity of the injury increased when the Par2 gene was mutated [92]. This study exemplifies direct tissue injury, in this case neurons, without immune system activation. However, in cases where the damage is immune-mediated, as can be seen below, Par2 aggravates the injury.

Par2 expression levels were elevated in samples from patients with multiple sclerosis, an autoimmune neurological disease [93]. Therefore, Noorbakhsh et al. induced experimental autoimmune encephalomyelitis (EAE) in mice and found that Par2KO ameliorated the disease phenotype [93]. In another EAE model, Göbel et al. demonstrated that Par2’s function in EAE induction is mediated by kallikrein processing, as Par2KO cells did not show a different response when kallikrein was added to their environment. Moreover, EAE score was highest in EAE MOG-induced WT mice, but significantly lower in kallikrein-deficient mice. However, when these KO mice were treated with a synthetic Par2 agonist, the EAE phenotype was regained [94].

In a different immune-mediated damage model using asphyxia-induced cardiac arrest by intravenous vecuronium administration, neurological damage was observed. Inhibition of mast cell tryptase significantly diminished the neurological injury, while a synthetic Par2 agonist exacerbated some of the damage, demonstrating the involvement of mast cell tryptase acting via Par2 activation [95]. Subsequently, Ocak et al. showed that the administration of a Par2 antagonist reduced the damage in this model [96].

Gut bacteria secrete proteases that activate Par2, increasing the neuronal excitability of vagal afferent neurons [97]. In their work, Pradhananga et al. investigated neuronal excitability rather than inflammatory or regenerative responses. This case demonstrates Par2 activation not induced in the immune system or the affected tissue, suggesting that there are additional aspects of Par2 modulation beyond the scope of our analysis.

### Pain

The discovery that Par2 affects pain signaling is well demonstrated by extensive studies from various fields. A direct such example is demonstrated, when the potent Par2 agonist, 2-aminothiazol-4-yl-LIGRL-NH_2_, was directly administered by intraplantar injection. The agonist induced robust hyperalgesic response, demonstrated by facial grimacing and mechanical hypersensitivity in mice. Interventions upstream to Par2 successfully inhibited these responses, supporting that the effect of the agonist was mediated only by Par2 [98]. In a subsequent study, the same group have developed a new antagonist, C781, capable of specifically inhibit this agonist by selectively blocking the β-arrestin/MAPK pathway [99]. This observation was further substantiated by the finding that application of the selective Par2 agonist 2at-LIGRL-NH2 (2AT) onto the dura elicited headache-related behavioral responses in WT mice, but not in Par2KO mice. Additionally, dural Par2 activation with 2AT led to priming to glyceryl trinitrate (GTN), while Par2KO mice exhibited no such priming response to GTN. Furthermore, behavioral responses to the endogenous protease neutrophil elastase, which can cleave and activate Par2, were assessed. Dural neutrophil elastase induced both acute responses and priming to GTN in WT mice, whereas Par2KO mice displayed no such responses [100].

In a study on aluminum ion ingestion-induced irritable bowel syndrome (IBS), it was observed that pain sensitivity was downregulated when Par2 was mutated. Mast cell activation, which is immune-mediated Par2 activation, was identified as the underlying mechanism. However, it remains unclear whether aluminum ions directly trigger IBS through Par2 activation or via another pathway involving mast cell stimulation, as Par2 is both activated by tryptase and induces inflammatory response in mast cells [101].

Hassler et al. demonstrated that a migraine-like mouse model is mediated by mast cell degranulation. Mast cells secrete tryptase, which activates Par2. The induction of a migraine-like phenotype was observed upon administration of a Par2 agonist, similar to the effects of a mast cell degranulator. Furthermore, the migraine-like symptoms induced by the mast cell degranulator were reduced by a Par2 antagonist and completely blocked in Par2KO mice [21]. In a subsequent study, these findings were translated into a potential pain relief therapy using a Par2-specific antibody, which was shown to block pain in general and alleviate migraine symptoms [102].

In a study on itch induced by mast cell cathepsin S (CatS) secretion, the ectopic administration of CatS and Par2 agonist both induced extensive itching. However, when Par2KO mice were administered together with these agents, itching was dramatically reduced [103].

Another study investigated pain elicited by a direct injury. Rats underwent paw incision, and their paw sensitivity was examined. Administration of a Par2 agonist increased nociceptive pain, heat, and mechanical responsiveness, while a Par2 antagonist alleviated these responses [1]. It is important to note that although a direct injury was induced, the regenerative response was not examined, and the authors focused solely on the perception of pain.

In an osteoarthritis mouse model, the injection of mast cells into a stimulated joint induced pain. This pain was reduced by a Par2 antagonist. When a Par2 agonist was injected instead of mast cells, the painful phenotype was regained, indicating that Par2 activation in the tissue is mediated by Par2 signaling [2].

### The respiratory system

Airway mucus is essential to many protective mechanisms in the respiratory system. It promotes clearance by washing different pollutants out of the airway tube. In addition, components of the airway mucus fight microbial agents with lysozymes, complement, and leukocytes present in it. In 2012, Lee et al. demonstrated that airway submucosal secretion is promoted by Par2 activation, improving airway protection. This was confirmed in mouse, pig, and human tissues, where isolated mucosal glands exposed to a synthetic Par2 agonist showed increased secretion [104]. However, a subsequent study by Asaduzzaman et al. showed that blocking Par2 with an antagonizing antibody (SAM-11) reduced mice’s allergic reaction induced by cockroach extract. When the extract was administered to mice without the antagonist, eosinophil numbers in the bronchoalveolar lavage (BAL) increased, along with higher collagen levels indicating scar formation in the airways [11]. Similarly, other studies demonstrated that Par2 antagonist treatment suppressed asthmatic phenotypes, including the appearance of reactive oxygen species and leukocyte infiltrations [10]. In accordance, when mice were infected with *Streptococcus pneumoniae*, Par2KO mice largely survived the infection, indicating that Par2 does not hinders a successful disposal of the bacteria. Nevertheless, in WT mice, Par2 aggravated inflammatory pneumonia induced by *S. pneumoniae* [9]. In another study simulating an asthmatic reaction to house dust mites (HDM) in mice, hematopoietic lineage mutation using BM transfer from Par2KO reduced the animals’ asthmatic reaction, indicating that Par2 worsens asthma induced by HDM [105]. Furthermore, it was found that Par2 induces the inflammatory effect by upregulating the IL-17 pathway in an HDM-induced asthma model [106]. Consistent with these findings, exposure to multi-walled carbon nanotubes and HDM extract synergistically increased lung inflammation, as assessed by histopathology and increased BAL, in both WT and Par2 KO mice. Moreover, both WT and Par2KO mice exhibited a similar increase in lung eosinophil chemokine CCL-11. However, Par2KO mice demonstrated significantly less airway fibrosis, accompanied by downregulation of the pro-fibrotic mediator arginase 1 [107].

In an asthmatic model induced by *Alternaria alternata*, a specific serine protease secreted by the fungi was sufficient to induce an asthmatic reaction in WT mice, while Par2KO mice were protected, indicating that *Alternaria*-induced asthma is propagated by Par2 activation [8]. In a subsequent study, a Par2 antagonist (C391) was proven to block Par2 signaling induced by the fungi [20]. A follow-up investigation showed that the antagonist C781, when given prophylactically, alleviated airway inflammation and mucus overproduction of the small airways in an acute allergen-challenged mouse model induced by *Alternaria alternata* [108]*.*

In a cystic fibrosis mouse model generated by the mutation of the β-epithelial Na^+^ channel (βENaC), Small et al. showed that introducing another mutation in the CatS gene alleviates the cystic fibrosis phenotype. Since Par2 is a CatS substrate acting downstream of CatS, blocking Par2 signaling with an antagonist in the βENaC null mutants dramatically reduced the disease phenotype [109], indicating that Par2 has an aggravating role in cystic fibrosis propagation.

Adding to the complexity, a recent work by Hagras and Kamel demonstrated that an asthmatic reaction was alleviated by the use of a Par2 agonist in guinea pigs, both in vivo and in vitro [110]. In contrast, Alaeida et al. showed that inhaled lipopolysaccharide (LPS) induces an asthmatic reaction via Par2 signaling through tryptase proteolytic activation secreted by mast cells [111].

Analyzing these reports based on the tissues in which Par2 is predominantly activated, the seeming contradictions can be resolved. When the immune system is activated, as in the cases of cockroach extract, HDM extract, *Alternaria alternata, Streptococcus pneumoniae,* and LPS, Par2 activation in immune cells leads to the aggravation of the asthmatic and inflammatory phenotypes [8–11, 20, 105–108, 111]. These asthma and inflammatory experiments were validated by the secretion of interleukins and chemokines, inflammatory markers, the presence of different leukocyte populations in the BAL, and the amount of secreted mucus. However, in the work by Hagras and Kamel, the evaluation of asthmatic reactions was determined by the diameter of the airway vessel. The bronchoalveolar radius represents the direct response of the lung tissue to Par2 signaling [110]. Therefore, since Par2 positively affects the tissue, Par2 activation appears to alleviate the disease. In the study by Lee et al., harvested tissues were removed from the animals [104]. Thus, it was not possible to recruit additional leukocytes that are not present in the tissue. Moreover, the examined phenotype was the secretion of mucus, which is an overall protective factor for the airways. In the case of the cystic fibrosis mouse model, a mutation in the βENaC gene prevents sodium reabsorption following mucus clearance, leading to the malfunction of an otherwise positive process for tissue healing [109]. Additionally, in this model, an aggravated immune response was observed when Par2 was activated by CatS.

Zhou et al. exploited the fact that Par2 aggravates the immune response to induce better vaccination efficacy in mice. The administration of influenza virosomes together with a Par2 agonist as a vaccination strategy resulted in improved survival compared to controls, the agonist alone, or virosomes alone. Moreover, when antibodies purified from vaccinated mice were isolated, the group that received influenza virosomes with the Par2 agonist generated the most efficient antibodies, providing the best protection against influenza. Additionally, there was increased T-cell activity, as adoptive transfer of CD8+ cells increased mice survival in the face of a lethal influenza challenge [112].

### The skin

Several studies have investigated the role of Par2 in skin-related conditions. In mouse models of atopic dermatitis, two different groups independently exposed Par2 overexpressing mice to HDM extract. Par2 overexpression led to extensive atopic dermatitis characterized by significant leukocyte infiltration, including lymphocytes, mast cells, and eosinophils [15, 17]. However, when mice with atopic dermatitis were topically treated with the Par2 antagonist FSLLRY, they showed milder epidermal hyperkeratosis and acanthosis, reduced leukocyte infiltration, angiogenesis, and scratching behavior [16].

Mast cell-derived tryptase, which activates Par2, was found to be involved in the secretion of thymic stromal lymphopoietin (TSLP), a cytokine associated with inflammatory dermatoses such as atopic dermatitis and some asthma cases. Redhu et al. showed that TSLP secretion is dependent on mast cells, specifically mast cell-secreted tryptase activated Par2. In vivo and in vitro samples from Par2KO mice did not induce TSLP-induced inflammation [113]. In a model of prolonged and sustained exposure to ultraviolet irradiation (UVB), mice treated with the Par2 antagonist GB83 exhibited a reduced inflammatory response and oxidative stress [114].

However, an additional investigation revealed that both low-dose trypsin and synthesized Par2 agonists notably accelerated the migration, adhesion, and proliferation of fibroblasts and macrophages in an in vitro setting. These cellular functions induced by trypsin were largely hindered by Par2 blockade, implying trypsin’s involvement via Par2 activation. Furthermore, in a non-inflammatory in vivo experiment conducted in rats, low-dose trypsin markedly expedited wound healing and regeneration while promoting collagen deposition [115]. The outcomes of this latter study, seemingly contradicting prior skin-related research, can be reconciled by our Par2 bi-modal activation theory. Here, the focus is on tissue regeneration, whereas the other studies primarily address immune-mediated mechanisms.

### Obesity

Obesity, type II diabetes, and a high fat diet induce low grade, basal, and chronic inflammation, characterized by increased levels of inflammatory cytokines and acute phase proteins [116–118]. In diet-induced obesity (DIO) models, Par2 promotes obesity, macrophage inflammation, and insulin resistance [119–121]. The protective role of Par2 in adipose inflammation and insulin resistance is evident as Par2KO ob/ob mice were largely shielded from these effects [122]. Analysis of liver samples from human patients revealed elevated Par2 expression in liver diseases, particularly non-alcoholic fatty liver disease (NAFLD) [123]. Consistent with these findings, Par2KO mice were resistant to steatosis induced by a high-fat diet (HFD) [123–125]. Notably, Par2KO inhibits adipogenesis, a regenerative pathway, as demonstrated by Park et al., who observed impaired adipocyte formation in Par2KO mice. The Par2 antagonist GB83 further hindered adipogenesis, whereas the Par2 agonist SLIGRL-NH2 accelerated adipogenesis. Both agonist and antagonist acted in concentration-dependent manners [126].

### Embryonic development

Embryonic development is often associated with regenerative processes, raising the expectation that Par2KO animals may exhibit impaired embryonic development. However, no developmental delays were observed in Par2KO pups [12, 23]. Nevertheless, examination of embryos at E13.5 and 18.5 revealed reduced fetal and placental weights [127]. These developmental delays, however, do not significantly impact on the size and weight of adult WT and Par2KO mice.

### Vision

In a study investigating the effect of *A. fumigatus* on corneal infection in mice, the authors discovered that inflammation induced by the fungi could be inhibited by a Par2 antagonist [128], supporting our hypothesis that activation of Par2 by the immune system exacerbates tissue damage, as observed in this model.

## Discussion

The Par2 GPCR has been shown to play significant roles in various diseases, inflammatory and regenerative processes, as well as many damage models. However, the actions of Par2 can be complex, sometimes exacerbating the phenotype and other times alleviating the damage. In this literature analysis, we aimed to map the role of Par2 based on its primary activation site within different tissues. Our analysis encompassed 128 recent manuscripts, spanning a range of tissues from the skin to the gut. Through this investigation, we have gained insights into the divergent effects of Par2 activation.

**Fig. 3:**
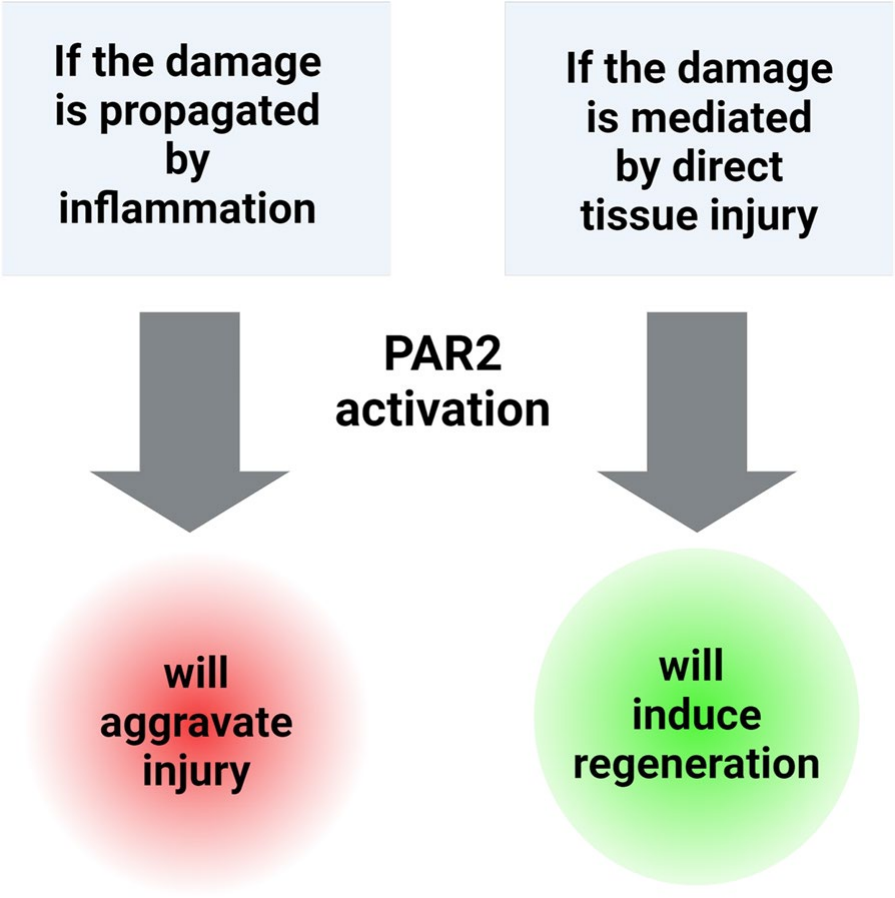
Dual role of Par2 depending on the site of activation

Our findings indicate that Par2 exhibits a detrimental role when its activation is initially induced in the immune system. This suggests that immune system-mediated activation of Par2 can contribute to the aggravation of injury or inflammation. However, when Par2 is not initially activated in the immune system, the injury triggers Par2 activation within the affected tissues themselves, leading to enhanced healing and regeneration. These observations are summarized in Fig. 3, illustrating the dual role of Par2 depending on the site of activation.

Based on our analysis, we propose that Par2, being a membrane-anchored receptor expressed on the cell surface, can be modulated to facilitate faster healing. Given the availability of potent Par2 agonists and antagonists, we suggest a strategic approach for drug administration. In cases of direct injury or poisoning, where tissue damage is the primary concern, Par2 activation using agonists may be a beneficial treatment approach. On the other hand, in situations involving acute immune or inflammatory incidents, Par2 inhibition using antagonists is recommended to mitigate the detrimental effects.

This study provides valuable insights into the complex role of Par2 in various tissues and conditions. By understanding the context-specific effects of Par2 activation, we can guide the development and application of therapeutic strategies targeting Par2. Further research is warranted to investigate the specific mechanisms underlying Par2-mediated effects and to explore the potential of modulating Par2 signaling pathways for therapeutic purposes.

In conclusion, our comprehensive analysis sheds light on the diverse functions of Par2 in different tissues and highlights the importance of considering the site of Par2 activation for therapeutic interventions. By unraveling the Par2 activation enigma, we propose a refined approach for utilizing Par2 agonists and antagonists to promote healing and mitigate immune-mediated tissue damage. These findings pave the way for future investigations and the development of innovative therapies aimed at harnessing the potential of Par2 modulation in clinical settings.

## Data Availability

All data generated during and/or analyzed during the current study are available from the corresponding author on reasonable request.

## Abbreviations

Par2: Protease-activated receptor 2
BAL: Bronchoalveolar lavage
BM: Bone marrow
cAMP: Cyclic adenosine monophosphate
CatS: Cathepsin S
CCl_4_: Carbon tetrachloride
ConA: Concanavalin A
DIO: Diet-induced obesity
DSS: Dextran sodium sulfate
EAE: Experimental autoimmune encephalomyelitis
eNOS: Endothelial nitric oxide synthase
GI: Gastrointestinal
GPCR: G-protein-coupled receptor
HBVX: Hepatitis B virus X
HDM: House dust mites
HFD: High-fat diet
IBD: Inflammatory bowel disease
IBS: Irritable bowel syndrome
KLK: Kallikrein
KO: Knockout
LAD: Left anterior descending artery
LDL: Low-density lipoprotein
LPS: Lipopolysaccharide
MHC: Major histocompatibility complex
NAFLD: Non-alcoholic fatty liver disease
NTN: Nephrotoxic serum nephritis
TL: Tethered ligand
TNBS: Trinitrobenzene sulphonic acid
TSLP: Thymic stromal lymphopoietin
UVB: Ultraviolet irradiation
VEGF: Vascular endothelial growth factor
WT: Wild-type
βENaC: β-Epithelial Na^+^ channel
